# Supplementary material for: LM-GVP: an extensible sequence and structure informed deep learning framework for protein property prediction
Source: Sci Rep. 2022 Apr 27;12:6832. doi: 10.1038/s41598-022-10775-y (PMC9046255; doi:10.1038/s41598-022-10775-y)
Supplement: Supplementary file 1 — Supplementary Information. [file 41598_2022_10775_MOESM1_ESM.pdf]

Supplementary Information for "LM-GVP: A Generalizable Deep Learning Framework for Protein Property Prediction from Sequence and Structure"

Zichen Wang[1*], Steven A. Combs[2*], Ryan Brand[1*], Miguel Romero Calvo[1], Panpan Xu[1], George Price[1], Nataliya Golovach[2], Emmanuel O. Salawu[1], Colby J. Wise[1], Sri Priya Ponnapalli[1✉], Peter M. Clark[2✉]

1: Amazon Machine Learning Solutions Lab, Amazon Web Services, Santa Clara, CA, USA
2: Janssen Biotherapeutics, The Janssen Pharmaceutical Companies of Johnson & Johnson, Spring House, PA, USA
*: equal contribution
✉: correspondence should be addressed to: S.P.P. (priyapo@amazon.com) and P.M.C. (PClark3@its.jnj.com)

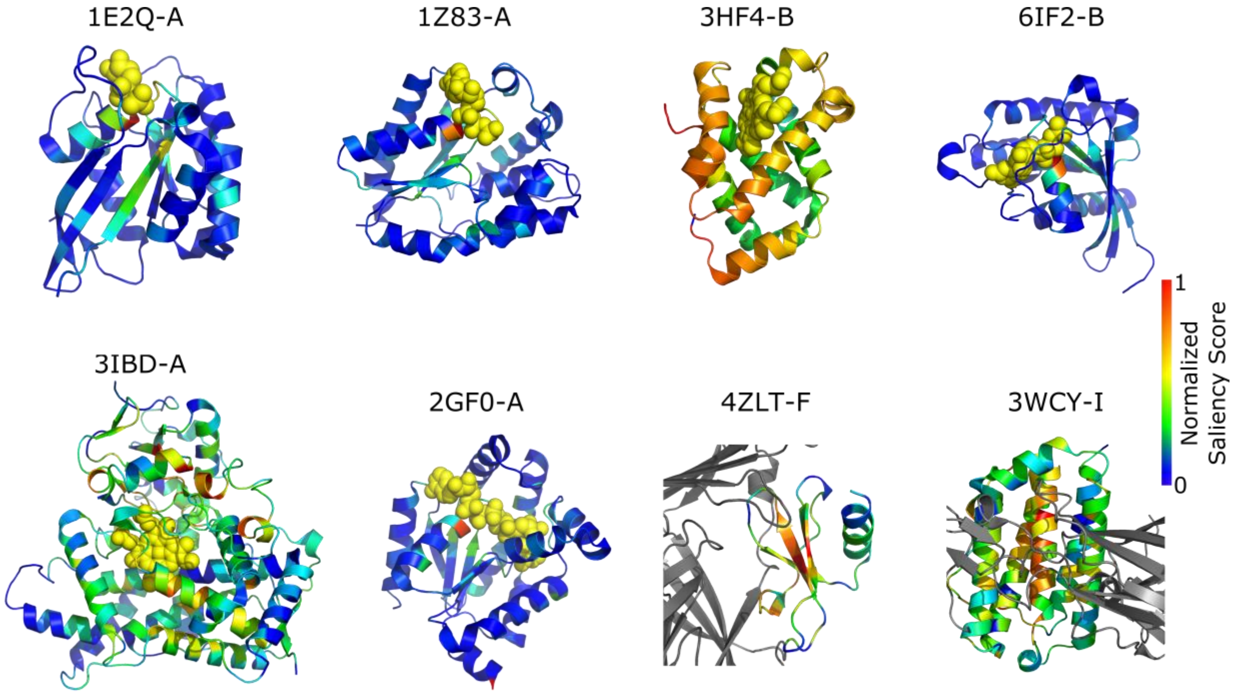

Figure S1. Identification of catalytic residues in enzymes based on saliency scores. For the first six complexes (i.e., 1E2Q-A to 2GF0-A), ligands are shown in yellow spheres while the residues of the receptors they bind to are colored based on saliency scores. In 4ZLT-F and 3WCY-I, the receptors are shown in grey and the residues of the protein ligands are colored based on saliency scores. All the saliency-score-based coloring are done such that the most salient residues are in shades of red and the least salient in shades of blue.

Table S1 AUPRs of GO terms with better predictability from sequence-only over structure-only model.

GO	GO term	task	Sequence-only	Structure-only	Sequence+Structure
**GO:0003707**	steroid hormone receptor activity	MF	1	0.066666667	1
**GO:0004308**	exo-alpha-sialidase activity	MF	1	0.1	1
**GO:0016997**	alpha-sialidase activity	MF	1	0.1	1
**GO:0016855**	racemase and epimerase activity, acting on amino acids and derivatives	MF	0.833333333	0.152631579	0.071794872
**GO:0036361**	racemase activity, acting on amino acids and derivatives	MF	0.75	0.233333333	0.069411765
**GO:0003777**	microtubule motor activity	MF	0.5034053	0.002587933	0.505068241
**GO:0003916**	DNA topoisomerase activity	MF	1	0.5	1
**GO:0003918**	DNA topoisomerase type II (double strand cut, ATP-hydrolyzing) activity	MF	1	0.5	1
**GO:0003796**	lysozyme activity	MF	1	0.505256648	0.854166667
**GO:0039633**	killing by virus of host cell	BP	1	0.510869565	1
**GO:0044659**	viral release from host cell by cytolysis	BP	1	0.512820513	1
**GO:0003968**	RNA-directed 5'-3' RNA polymerase activity	MF	0.480903475	0.019437661	0.346347559
**GO:0047661**	amino-acid racemase activity	MF	0.642857143	0.2	0.093073593
**GO:0008238**	exopeptidase activity	MF	0.754598846	0.332999142	0.831611136
**GO:0019030**	icosahedral viral capsid	CC	0.505780347	0.091666667	1
**GO:0008237**	metallopeptidase activity	MF	0.730667377	0.347115767	0.768600405
**GO:0010181**	FMN binding	MF	0.543060779	0.168609832	0.531734474
**GO:0034061**	DNA polymerase activity	MF	0.503498652	0.132337175	0.416682176
**GO:0004180**	carboxypeptidase activity	MF	0.657264616	0.308982453	0.730095663
**GO:0097747**	RNA polymerase activity	MF	0.523292297	0.178760823	0.612304907
**GO:0034062**	5'-3' RNA polymerase activity	MF	0.528279096	0.184061032	0.613787522
**GO:0008242**	omega peptidase activity	MF	0.839099489	0.499642491	0.772809194
**GO:0008235**	metalloexopeptidase activity	MF	0.798551552	0.469072297	0.83554996
**GO:0036459**	thiol-dependent ubiquitinyl hydrolase activity	MF	0.900445144	0.573133386	0.801567364
**GO:0005747**	mitochondrial respiratory chain complex I	CC	0.801212998	0.48022218	0.983333333

Table S2 AUPRs of GO terms with better predictability from structure-only over sequence-only model.

GO	GO term	task	Sequence-only	Structure-only	Sequence+Structure
**GO:0019031**	viral envelope	CC	0.000350018	1	0.1
**GO:0005839**	proteasome core complex	CC	0.080430341	0.996732026	0.987272102
**GO:0009538**	photosystem I reaction center	CC	0.1	1	1
**GO:0015671**	oxygen transport	BP	0.008422677	0.874088713	0.907005539
**GO:0005833**	hemoglobin complex	CC	0.032878991	0.875392465	0.878095975
**GO:0006662**	glycerol ether metabolic process	BP	0.037785574	0.833956562	0.834029301
**GO:0010499**	proteasomal ubiquitin-independent protein catabolic process	BP	0.014935254	0.80367336	0.858815872

**GO:0034987**	immunoglobulin receptor binding	MF	0.018421424	0.805555556	0.75
**GO:0018904**	ether metabolic process	BP	0.03259338	0.764446168	0.784910714
**GO:0030288**	outer membrane-bounded periplasmic space	CC	0.037568365	0.746115405	0.834994734
**GO:0046940**	nucleoside monophosphate phosphorylation	BP	0.140335496	0.836811004	0.843889519
**GO:0019877**	diaminopimelate biosynthetic process	BP	0.185185185	0.833333333	0.392857143
**GO:0042611**	MHC protein complex	CC	0.236180853	0.868707483	0.961734694
**GO:0042597**	periplasmic space	CC	0.044804497	0.665662494	0.829091316
**GO:0043190**	ATP-binding cassette (ABC) transporter complex	CC	0.029957143	0.633781889	0.274275519
**GO:0016833**	oxo-acid-lyase activity	MF	0.134657277	0.731448413	0.717261905
**GO:0034219**	carbohydrate transmembrane transport	BP	0.0416284	0.608886678	0.64168028
**GO:0098533**	ATPase dependent transmembrane transport complex	CC	0.037442034	0.603484875	0.22486509
**GO:0015144**	carbohydrate transmembrane transporter activity	MF	0.257598442	0.821706994	0.891284132
**GO:0010257**	NADH dehydrogenase complex assembly	BP	0.02067914	0.584309815	0.850965406
**GO:0032981**	mitochondrial respiratory chain complex I assembly	BP	0.021210449	0.578296893	0.850598099
**GO:0030313**	cell envelope	CC	0.099814634	0.651919957	0.874100818
**GO:0015669**	gas transport	BP	0.020696337	0.559137371	0.587183775
**GO:0008643**	carbohydrate transport	BP	0.047395237	0.573559466	0.689976611
**GO:0008218**	bioluminescence	BP	0.000827713	0.501315789	0.125773994

Table S3 AUPRs of GO terms with better predictability from sequence-only over LM-GVP model.

GO	GO term	task	Sequence-only	Structure-only	Sequence+Structure
**GO:0016855**	racemase and epimerase activity, acting on amino acids and derivatives	MF	0.833333333	0.152631579	0.071794872
**GO:0036361**	racemase activity, acting on amino acids and derivatives	MF	0.75	0.233333333	0.069411765
**GO:0047661**	amino-acid racemase activity	MF	0.642857143	0.2	0.093073593
**GO:0099094**	ligand-gated cation channel activity	MF	0.611661857	0.344005535	0.406452375
**GO:0003796**	lysozyme activity	MF	1	0.505256648	0.854166667
**GO:0003968**	RNA-directed 5'-3' RNA polymerase activity	MF	0.480903475	0.019437661	0.346347559
**GO:0030682**	mitigation of host defenses by symbiont	BP	0.254083622	0.017744474	0.12784732
**GO:0018024**	histone-lysine N-methyltransferase activity	MF	0.551334781	0.412385246	0.443394616
**GO:0042178**	xenobiotic catabolic process	BP	0.13221182	0.008117095	0.029824954
**GO:0046173**	polyol biosynthetic process	BP	0.173973669	0.014808723	0.073239294
**GO:0036459**	thiol-dependent ubiquitinyl hydrolase activity	MF	0.900445144	0.573133386	0.801567364

**GO:0016894**	endonuclease activity, active with either ribo- or deoxyribonucleic acids and producing 3'-phosphomonoesters	MF	0.437391304	0.208403674	0.343305286
**GO:0034061**	DNA polymerase activity	MF	0.503498652	0.132337175	0.416682176
**GO:0003684**	damaged DNA binding	MF	0.261815821	0.059978214	0.180120532
**GO:0016846**	carbon-sulfur lyase activity	MF	0.464867425	0.357905779	0.385106564
**GO:0016829**	lyase activity	MF	0.637267563	0.408398336	0.560307538
**GO:0101005**	ubiquitinyl hydrolase activity	MF	0.9061601	0.669037003	0.834046481
**GO:0003774**	motor activity	MF	0.262622343	0.006011441	0.191448181
**GO:0003887**	DNA-directed DNA polymerase activity	MF	0.461435822	0.214752411	0.394313091
**GO:0004550**	nucleoside diphosphate kinase activity	MF	0.924569189	0.91808021	0.857487674
**GO:0008242**	omega peptidase activity	MF	0.839099489	0.499642491	0.772809194
**GO:0016668**	oxidoreductase activity, acting on a sulfur group of donors, NAD(P) as acceptor	MF	0.671214877	0.683386425	0.61191512
**GO:0006586**	indolalkylamine metabolic process	BP	0.082020265	0.013229232	0.024697572
**GO:0046209**	nitric oxide metabolic process	BP	0.090468994	0.022600092	0.034714297
**GO:0016830**	carbon-carbon lyase activity	MF	0.495853967	0.412075369	0.440608859

Table S4 AUPRs of GO terms with better predictability from structure-only over LM-GVP model.

GO	GO term	task	Sequence-only	Structure-only	Sequence+Structure
**GO:0019031**	viral envelope	CC	0.000350018	1	0.1
**GO:0008800**	beta-lactamase activity	MF	0.03030303	0.5	0.010416667
**GO:0019877**	diaminopimelate biosynthetic process	BP	0.185185185	0.833333333	0.392857143
**GO:0098533**	ATPase dependent transmembrane transport complex	CC	0.037442034	0.603484875	0.22486509
**GO:0008218**	bioluminescence	BP	0.000827713	0.501315789	0.125773994
**GO:0043190**	ATP-binding cassette (ABC) transporter complex	CC	0.029957143	0.633781889	0.274275519
**GO:0044800**	multi-organism membrane fusion	BP	0.010746946	0.334111028	0.019165462
**GO:0044803**	multi-organism membrane organization	BP	0.009448908	0.334104649	0.021865327
**GO:0039663**	membrane fusion involved in viral entry into host cell	BP	0.010617676	0.334095174	0.023175529
**GO:0016730**	oxidoreductase activity, acting on iron-sulfur proteins as donors	MF	0.168741355	0.502066116	0.201149425
**GO:0036338**	viral membrane	CC	0.000372162	0.333333333	0.090909091
**GO:0016661**	oxidoreductase activity, acting on other nitrogenous compounds as donors	MF	0.062531551	0.385346097	0.145582118
**GO:0046654**	tetrahydrofolate biosynthetic process	BP	0.06837484	0.510535654	0.286644877
**GO:0070125**	mitochondrial translational elongation	BP	0.006161219	0.421238433	0.253132782
**GO:0022835**	transmitter-gated channel activity	MF	0.8625	1	0.834482759

**GO:0022824**	transmitter-gated ion channel activity	MF	0.876923077	1	0.834482759
**GO:0009396**	folic acid-containing compound biosynthetic process	BP	0.05354273	0.346903804	0.182038821
**GO:0036361**	racemase activity, acting on amino acids and derivatives	MF	0.75	0.233333333	0.069411765
**GO:0050661**	NADP binding	MF	0.160266767	0.421026165	0.257654266
**GO:0005504**	fatty acid binding	MF	0.082495375	0.418819313	0.266067619
**GO:0009240**	isopentenyl diphosphate biosynthetic process	BP	0.079429465	0.379492632	0.23113727
**GO:0046490**	isopentenyl diphosphate metabolic process	BP	0.094532576	0.365055717	0.229154951
**GO:0097529**	myeloid leukocyte migration	BP	0.070736734	0.393498494	0.275215605
**GO:0047661**	amino-acid racemase activity	MF	0.642857143	0.2	0.093073593
**GO:0072529**	pyrimidine-containing compound catabolic process	BP	0.040578565	0.169616023	0.063848784

Table S5 AUROC quantifying the agreement between saliency scores from LM-GVP and known active sites responsible for respective MF.

Protein	GO-MF	AUROC
**1ZBD-A**	GTP binding	0.63258
**4ARZ-A**	GTP binding	0.83401
**2WKQ-A**	GTP binding	0.87747
**1J2J-A**	GTP binding	0.66708
**3RAP-R**	GTP binding	0.68174
**2A5F-A**	GTP binding	0.79464
**1E2Q-A**	ATP binding	0.78003
**1UA2-A**	ATP binding	0.81737
**3MN5-A**	ATP binding	0.68845
**2QXL-A**	ATP binding	0.73843
**1C0F-A**	ATP binding	0.74984
**2PAA-A**	ATP binding	0.53931
**4AAR-A**	ATP binding	0.76359
**2RCM-A**	Heme binding	0.73864
**1KQG-C**	Heme binding	0.67094
**1SY7-A**	Heme binding	0.79512
**2W0A-A**	Heme binding	0.70675
**1A4E-A**	Heme binding	0.78301
**2IAG-A**	Heme binding	0.74512
**1A9W-E**	Heme binding	0.62762
**2QRW-A**	Heme binding	0.58025
